# Structure‐Based Demystification of Radical Catalysis by a Coenzyme B_12_ Dependent Enzyme—Crystallographic Study of Glutamate Mutase with Cofactor Homologues

**DOI:** 10.1002/anie.202208295

**Published:** 2022-07-21

**Authors:** Karl Gruber, Vanessa Csitkovits, Andrzej Łyskowski, Christoph Kratky, Bernhard Kräutler

**Affiliations:** ^1^ Institute of Molecular Biosciences University of Graz Humboldtstraße 50 8010 Graz Austria; ^2^ BioTechMed-Graz 8010 Graz Austria; ^3^ Field of Excellence “BioHealth” University of Graz 8010 Graz Austria; ^4^ Institute of Organic Chemistry University of Innsbruck Innrain 80/82 6020 Innsbruck Austria; ^5^ Center of Molecular Biosciences (CMBI) University of Innsbruck 6020 Innsbruck Austria; ^6^ Present address: Department of Biotechnology and Bioinformatics Rzeszów University of Technology al. Powstańców Warszawy 12 35-959 Rzeszów Poland

**Keywords:** Adenosyl Radical, Cobalamin, Enzyme Inhibitors, Radical Rearrangement, Transition State Mimics

## Abstract

Catalysis by radical enzymes dependent on coenzyme B_12_ (AdoCbl) relies on the reactive primary 5′‐deoxy‐5′adenosyl radical, which originates from reversible Co−C bond homolysis of AdoCbl. This bond homolysis is accelerated roughly 10^12^‐fold upon binding the enzyme substrate. The structural basis for this activation is still strikingly enigmatic. As revealed here, a displaced firm adenosine binding cavity in substrate‐loaded glutamate mutase (GM) causes a structural misfit for intact AdoCbl that is relieved by the homolytic Co−C bond cleavage. Strategically interacting adjacent adenosine‐ and substrate‐binding protein cavities provide a tight caged radical reaction space, controlling the entire radical path. The GM active site is perfectly structured for promoting radical catalysis, including “negative catalysis”, a paradigm for AdoCbl‐dependent mutases.

## Graphical abstract

Glutamate mutase (GM) catalyses the interconversion of glutamate and 3‐methylaspartate. It binds its B_12_‐cofactor AdoCbl with an essential misfit for its Ado‐group, liberating the clamped down aggressive Ado‐radical in a tight reaction space to even promote “negative catalysis”. A “simply stretched” AdoCbl‐homolog, in contrast, fits GM, freezing up the intriguing functional structure of this enzyme.

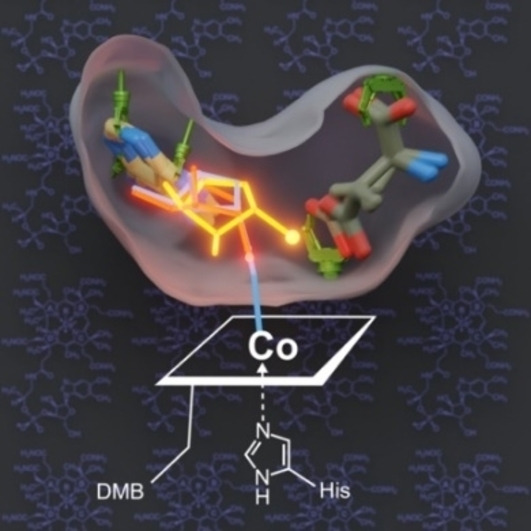

## Introduction

The exceptional, transformative capacities of radical processes enrich Nature's molecular machinery with unparalleled chemistry.^[1]^ However, the reactivity of radical species^[2]^ also calls for their metabolic submission to precise control.[^1c^, ^1d^, ^3^] Radical enzymes dependent upon coenzyme B_12_ (AdoCbl, adenosylcobalamin), first identified over six decades ago,^[4]^ play exemplary roles in Nature^[5]^ and remain in the “scientific spotlight”.^[6]^ In such enzymes, the organometallic B_12_‐cofactor AdoCbl acts as a reversibly functioning radical source,^[7]^ setting free the highly reactive primary 5′‐deoxy‐5′‐adenosyl radical (Ado‐radical).^[8]^ Like other radical enzymes, AdoCbl‐dependent enzymes rely on i) the substrate‐initiated generation of the catalytic Ado‐radical and ii) the tight control of the subsequent, rapid radical reaction steps, such as H‐atom transfer and radical isomerisations.^[6]^ AdoCbl‐dependent enzymes are, thus, considered exemplary for the operation of “negative catalysis”,^[3b]^ despite many still lacking details of their puzzling biochemical paths.[^6^, ^9^]

**Figure 1:**
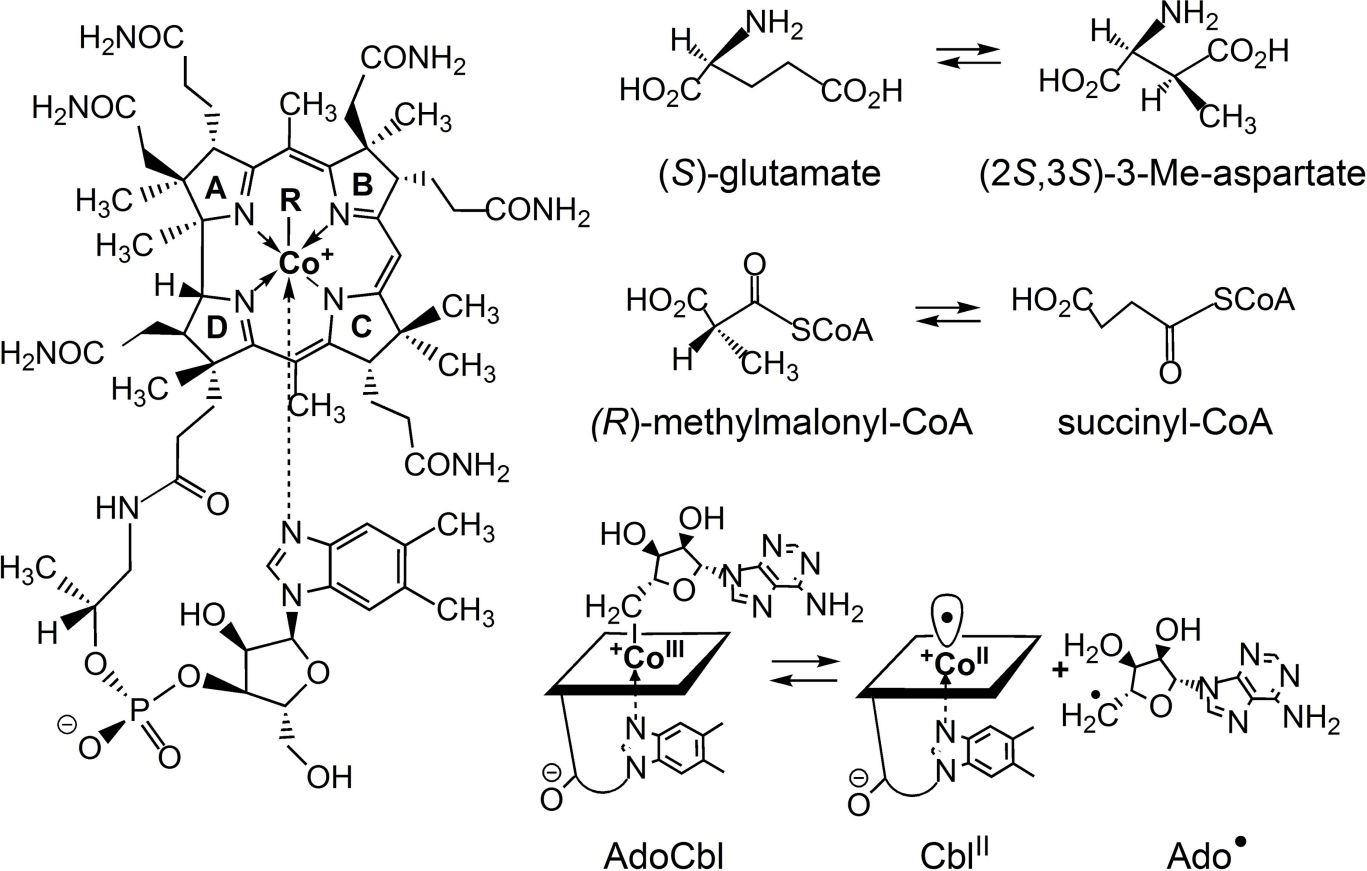
Left: Structural formula of the cobalamins vitamin B_12_ (CNCbl, R=CN), coenzyme B_12_ (AdoCbl, R=5′‐deoxy‐5′‐adenosyl), methylcobalamin (MeCbl, R=CH_3_), cob(II)alamin (Cbl^II^, R=e^−^). Right: The carbon skeleton isomerisations catalysed by GM and MCM, and depiction of the Co−C bond homolysis of AdoCbl that generates the catalytically active 5′‐deoxy‐5′‐adenosyl‐radical (Ado⋅) and Cbl^II^ reversibly.

Studies of methylmalonyl‐CoA mutase (MCM), which achieves the isomerisation of (*R*)‐methylmalonyl‐CoA to succinyl‐CoA,^[6b]^ and of glutamate mutase (GM),[^1c^, ^4^, ^10^] which interconverts the amino acid (*S*)‐glutamate and its isomer (2*S*,3*S*)‐3‐methylaspartate (Figure 1), have provided pioneering insights into the biochemical mechanisms, structures, and modes of action of AdoCbl‐dependent enzymes.[^6a^, ^6b^, ^10^, ^11^] A fascinating feature of these[^6d^, ^10^, ^12^] and other AdoCbl‐dependent enzymes[^1a^, ^6b^, ^6c^, ^13^] is the roughly 10^12^‐fold acceleration of the homolysis of the ca. 31 kcal mol^−1^ strong Co−C bond of AdoCbl, induced by the binding of the enzyme substrates. The homolytic cleavage of the Co−C bond[^7^, ^14^] of AdoCbl furnishes not only the still elusive Ado‐radical^[8]^ but also the highly effective radical trap cob(II)alamin (Cbl^II^) (Figure 1).^[15]^ This circumstance endows AdoCbl‐dependent enzymes with a highly reactive Ado‐radical, whose transient existence is coupled to exceedingly fast reactions, including its combination with Cbl^II^ to regenerate intact AdoCbl by Co−C bond formation.[^6b^, ^16^]

**Figure 2:**
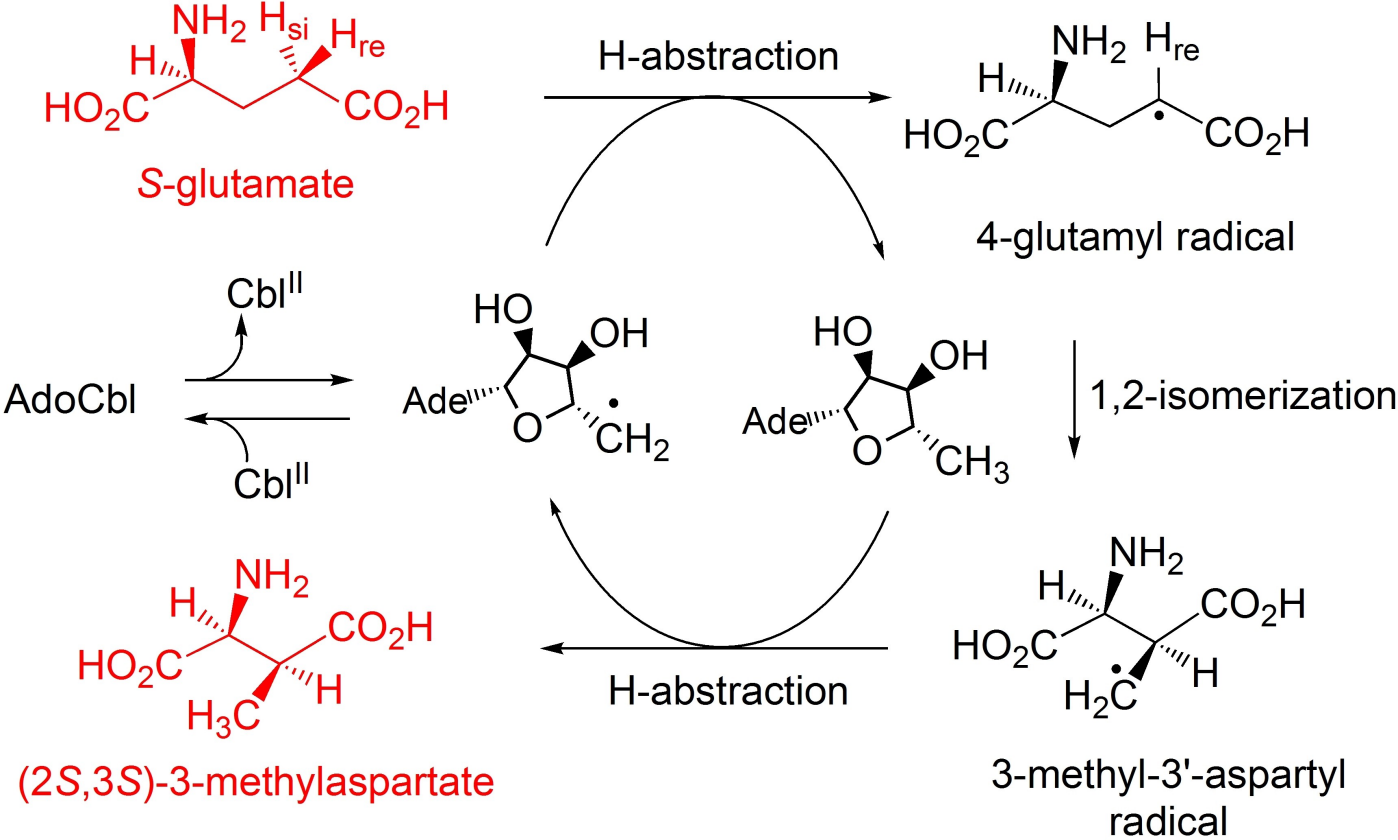
Key radical steps in the reversible isomerisation catalysed by the AdoCbl‐dependent GM depicted in the direction from (*S*)‐glutamate to (2*S*,3*S*)‐3‐methylaspartate.

The AdoCbl‐dependent carbon skeleton mutases display a common basic architecture featuring a coenzyme B_12_‐binding Rossmann fold, a triosephosphate‐isomerase (TIM)‐barrel making most contacts with the substrate molecules^[11]^ and AdoCbl bound at, or near, the interface[^12a^, ^17^] in the “base‐off/His‐on” form, where a histidine residue of the Rossmann fold domain coordinates to the cobalt‐centre.^[18]^ As discovered in MCM^[19]^ and similarly found in other acyl‐CoA mutases,^[20]^ substrate‐binding induces a reorientation of the B_12_‐binding vs. the TIM barrel domains.^[11]^ While the detailed structural changes caused by substrate‐binding have not been elucidated with GM,^[12a]^ studies with this enzyme provided first structural insights into how the Ado‐radical was generated and “shuttled” to a position close to the substrate.^[21]^ The process catalysed by GM^[12a]^ assigns the enzyme‐generated Ado‐radical the specific function of abstracting (in the direction from (*S*)‐glutamate to (2*S*,3*S*)‐3‐methylaspartate) the pro‐S H‐atom from the 4‐position of (*S*)‐glutamate, furnishing 5′‐deoxyadenosine and a 4‐glutamyl radical (see Figure 2).^[22]^ The latter radical isomerises to a 3‐methyl‐3′‐aspartyl radical, which re‐abstracts an H‐atom from C5′ of deoxyadenosine producing (2*S*,3*S*)‐3‐methylaspartate and the Ado‐radical, which recombines with Cbl^II^ to regenerate AdoCbl.[^10^, ^12a^] However, to this date, the precise structural basis of both the exceptional substrate‐induced acceleration of Co−C bond homolysis of AdoCbl and the subsequent enzyme‐controlled radical reaction is still poorly established.[^10^, ^11^, ^13b^, ^21^]

The initial X‐ray structural analysis of AdoCbl bound to substrate‐loaded GM^[21]^ has provided an insightful model for the path of the Ado‐radical from the cobalt‐corrin to the substrate, a suitable model also for the radical enzyme MCM[^6d^, ^19^, ^23^] and other natural acyl‐CoA mutases.^[20]^ In substrate‐loaded GM‐AdoCbl, two ribose conformations of the adenosyl group were detected, laying out a precise conformational restructuring of the tightly bound 5′‐deoxy‐5′‐adenosyl radical (Ado‐radical) by a pseudo‐rotation of the ribose moiety, the “radical shuttling” pathway. In GM, this Ado‐reorientation was correlated with the substrate‐induced repositioning of its C5′ from the cobalt centre to a “radical‐associated (r_a_) conformation” with a Co−C distance of 3.2 Å and to a “radical‐dissociated (r_d_) conformation” with a Co−C distance of about 4.5 Å. The “radical shuttling” reflects cleavage of the Co−C bond and a further roughly 1.7 Å movement of C5′ to near the bound substrate for the critical activation by H‐atom abstraction.

**Figure 3:**
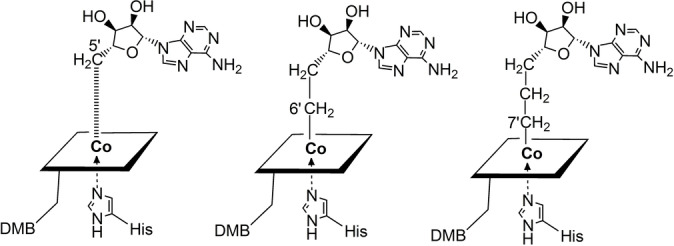
Symbolic representations of AdoCbl and AdoCbl‐homologues bound to GM base‐off/His‐on, where “base” refers to 5,6‐dimethylbenzimidazole (DMB). Left: AdoCbl is bound by substrate‐loaded GM and critically activated with elongation or cleavage of the Co−C bond. Centre and right: The two “stretched” covalent homologues of coenzyme B_12_, homocoenzyme B_12_ (AdoMeCbl, 5′‐deoxy‐5′‐adenosylmethyl‐cobalamin) and bis‐homocoenzyme B_12_ (AdoEtCbl, 7′[5′‐deoxy‐5′‐ethyladenosyl)‐cobalamin) are bound with their Co−C bonds intact.

**Figure 4:**
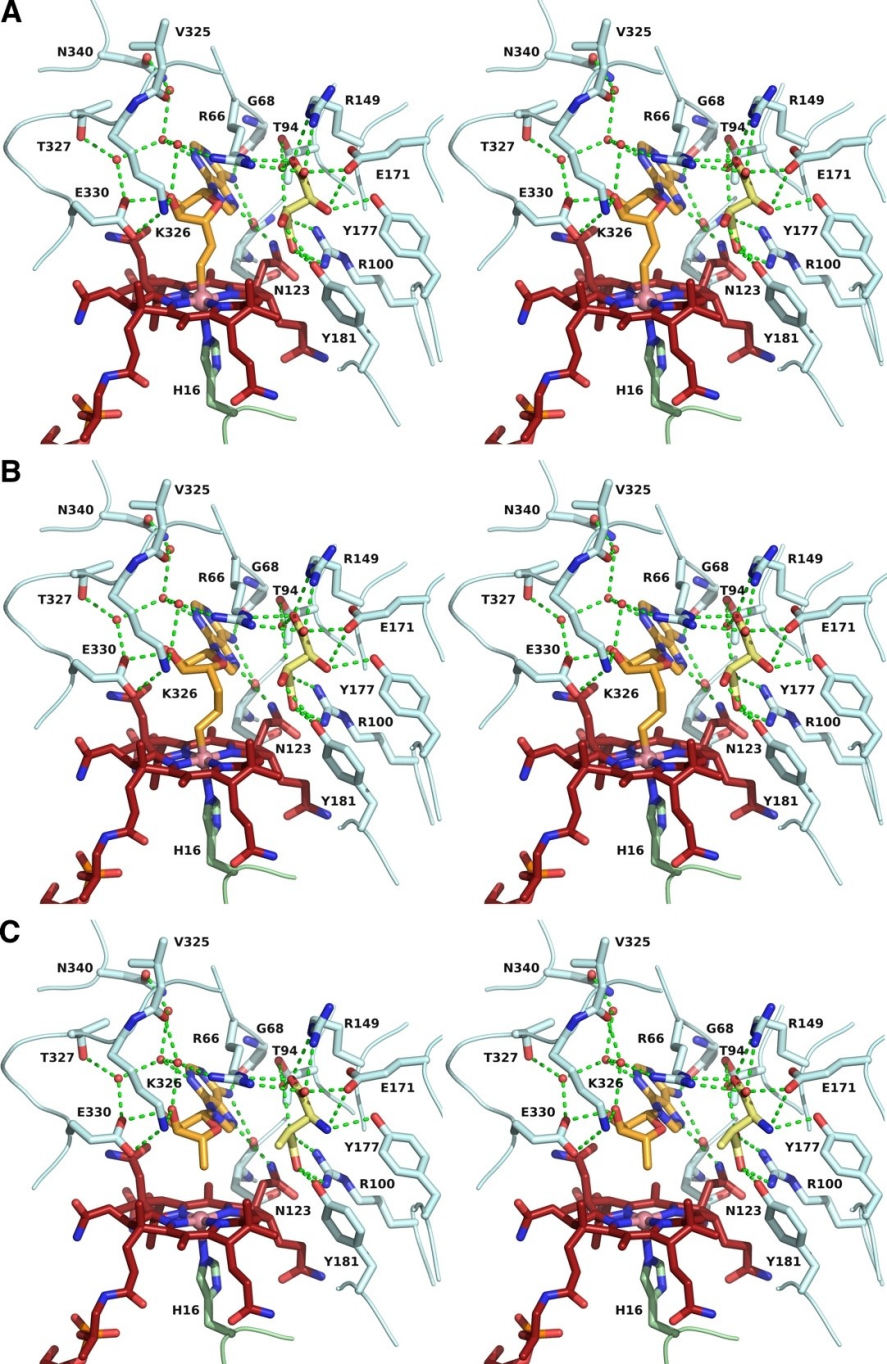
Stereo‐views of the active site of tartrate‐loaded GM, A) with AdoMeCbl bound, B) with AdoEtCbl bound, and C) stereo‐view of the active site of 3‐methylaspartate‐loaded GM (PDB‐code: 1I9C)^[21]^ with AdoCbl bound in r_a_‐conf (for other important combinations see Supporting Information Figure S3). Amino acid side chains, cofactors, and ligands are shown as sticks. Portions of the main chain of GM are shown as a ribbons‐representation. Residues from the ϵ‐ and the σ‐subunits are shown with light cyan or light green carbon atoms, respectively. The cobalamin is shown in dark red, with the “upper” ligand shown in orange. Tartrate and methylaspartate molecules are coloured yellow. Water molecules are depicted as small red spheres. Hydrogen bonding interactions are shown as green dashed lines.

An increased distance between the cobalt centre and the previously bound C5′ of the Ado‐moiety has been a consistent crystallographic observation with substrate‐loaded GM^[21]^ and with a range of other AdoCbl‐dependent enzymes.[^6c^, ^6d^, ^19^, ^20^, ^23^] However, the cobalt‐corrin moiety of the bound activated AdoCbl of the carbon skeleton mutases features a notably similar structure as in AdoCbl^[24]^ and as observed in its fragment Cbl^II^.^[15]^ Hence, these biostructural studies have failed to support a “butterfly” conformational deformation of the corrin ring as the key mode of AdoCbl‐activation.^[7]^ Instead, they are in line (see, e.g.[^6c^, ^6d^, ^21^]) with the hypothesis of strong protein‐binding of the largely separated fragments, Cbl^II^ and Ado‐radical, as the critical structural component of the activation of AdoCbl.^[15]^ We have set out to test this AdoCbl‐activation hypothesis with two “stretched” AdoCbl‐homologues, designed and synthesised as potential structural mimics of “activated” AdoCbl, provisionally named “homocoenzyme B_12_” (AdoMeCbl) and “bis‐homocoenzyme B_12_” (AdoEtCbl) (Figure 3).^[25]^ Consequently, the structures of GM reconstituted with AdoMeCbl or AdoEtCbl were expected to provide structural insights into the mode of activation of the bound AdoCbl for homolysis of its Co−C bond. We describe here the high‐resolution crystal structures of GM from *Clostridium cochlearium*, reconstituted with AdoMeCbl and AdoEtCbl and the pseudo‐substrate (*S*,*S*)‐tartrate (see Figure 4 and Supporting Information Figure S1, and Tables S1 and S2).

## Results and Discussion

As previously observed,[^21^, ^26^] GM forms a hetero‐tetramer consisting of two copies of the larger ϵ‐subunit (an (αβ)_8_ TIM‐barrel) and the smaller B_12_‐binding σ‐subunit, showing a typical Rossman fold.^[11]^ The ϵ‐subunit harbours the substrate‐binding site comprising three arginine residues (the “arginine claw”) that undergo strong polar binding interactions with the carboxylate groups of an (*S*,*S*)‐tartrate ion.[^21^, ^26^] The structures of the corrin moieties of the bound AdoCbl‐homologues AdoMeCbl and AdoEtCbl closely match the corresponding parts of bound AdoCbl itself (see Figure 4 and Supporting. Information Figure S1). AdoMeCbl and AdoEtCbl are bound in a base‐off/His‐on form, with distances between cobalt and the coordinating histidine imidazole of 2.21 Å and 2.17 Å (Supporting Information Table S2), respectively, similar to GM‐bound AdoCbl (2.22 Å)^[21]^ and free AdoMeCbl (2.19 Å).^[25a]^

**Figure 5:**
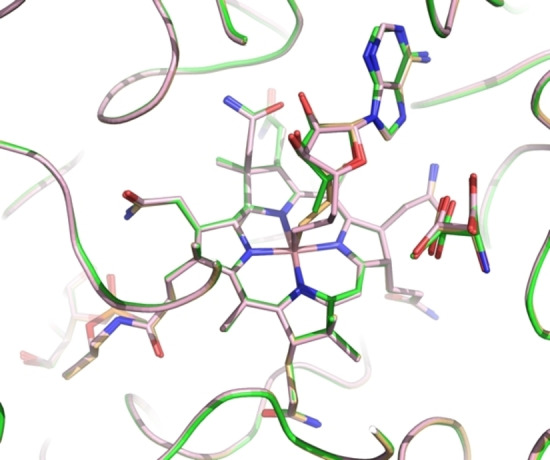
Superposition of active site structures of GM reconstituted with AdoCbl (green, r_a_‐conf), AdoMeCbl (orange), and AdoEtCbl (pink), highlighting stick‐models of the bound homologous AdoCbls, the substrate 3‐methylaspartate (green), and the pseudo‐substrate tartrate (pink).

The two structures displayed remarkably similar protein components that matched the previously determined GM structures[^21^, ^26^] with pairwise Cα root‐mean‐square‐deviations of 0.1 to 0.2 Å (Supporting Information Figure S2). Hence, the superposition of the structures of GM reconstituted with AdoCbl, AdoMeCbl or AdoEtCbl also led to an almost perfect structural match of the bound tartrate molecules and base‐off/His‐on Cbls, specifically including their entire adenosine units (Figures 4 and 5, Supporting Information Figure S5). In both new structures, the electron density for the “stretched” AdoCbl‐homologues indicated an intact bond between the cobalt ion and the terminal carbons C6′ or C7′ of the 5′‐methyl‐ or 5′‐ethyl‐adenosyl ligands of AdoMeCbl and AdoEtCbl, respectively (Supporting Information Figure S4). The refined Co−C bond lengths at 2.07 Å or 1.98 Å for the bound AdoMeCbl and AdoEtCbl, respectively, were somewhat longer or slightly shorter than the 2.00 Å observed in the crystal structures of AdoCbl^[24]^ and AdoMeCbl,^[25a]^ but in stark contrast to distances of >3.2 Å observed between cobalt and C5′ of the adenosyl ligand in GM reconstituted with AdoCbl.^[21]^ The distances from Co to C5′ of the Ado moieties of the bound AdoMeCbl and AdoEtCbl were 3.2 Å and 4.2 Å, strikingly comparable to the distances between the Co‐atom and C5′ in the two observed structures of “activated” AdoCbl in the crystal structure of GM reconstituted with AdoCbl.^[21]^

**Figure 6:**
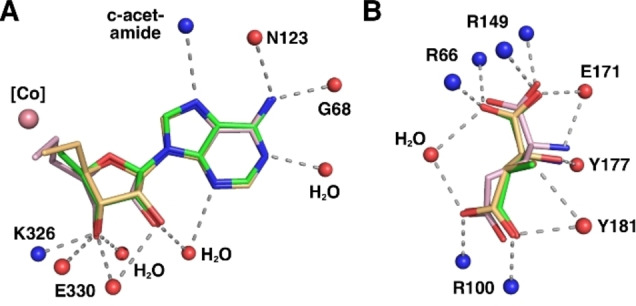
Tight binding of Ado‐ligands and (pseudo)substrates to GM. A) Schematic representation of the conserved interactions of the Ado‐, AdoMe‐ and AdoEt‐ligand of AdoCbl and its homologues within the adenosyl binding site (colour code as in Figure 5). B) Schematic representation of the common H‐bonding interactions of (*S*)‐glutamate (pink), (2*S*,3*S*)‐3‐methylaspartate (green), or (*S,S*)‐tartrate (orange) within the substrate‐binding site.

**Figure 7:**
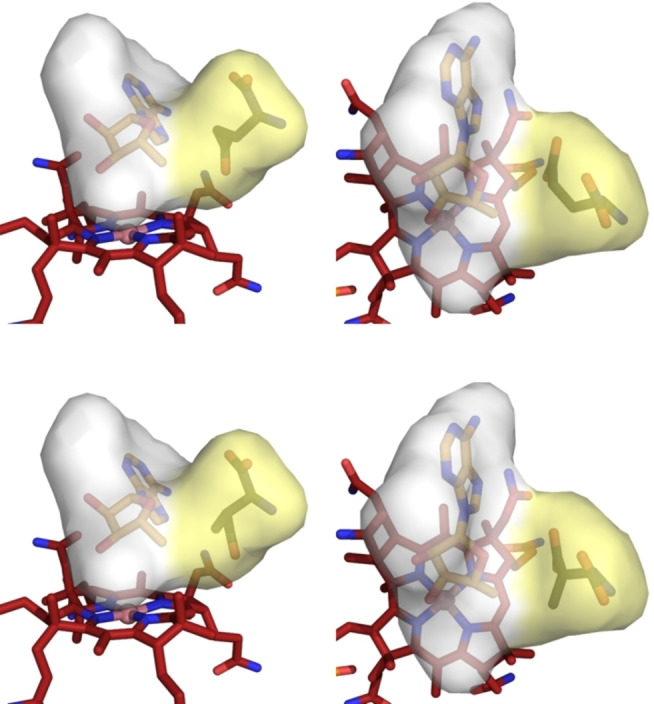
Substrate‐loaded GM‐AdoCbl features a rigid caged radical reaction space (CRRS) next to the bound B_12_‐cofactor. The CRRS consists of the mutually interacting and adjacent adenosine‐ and substrate‐binding cavities (ABC and SBC, coloured faint grey and faint yellow, respectively). The cavities were calculated using the program CavMan (innophore.com). It stabilises the bound “activated” AdoCbl and provides a firm grip for substrates, (radical) intermediates, and products, ensuring tight control (“negative catalysis”) for the radical process of GM. Shown here are two views of GM‐AdoCbl (as derived from the crystal structure)^[21]^ with “activated” AdoCbl featuring a largely separated Ado‐moiety in the r_d_‐conf and with the substrates (*S*)‐glutamate (top) or with (2*S*,3*S*)‐3‐methylaspartate (bottom) bound (see Supporting Information Figure S14 for corresponding views with Ado in r_a_‐conf).

The adenosyl moiety of the bound Cbls and its protein environment are positioned at a nearly indistinguishable location in substrate‐bound GM reconstituted with AdoCbl (GM‐AdoCbl), AdoMeCbl (GM‐AdoMeCbl), or AdoEtCbl (GM‐AdoEtCbl) (Supporting Information Figure S5), clearly defining a firm H‐bonding “adenosine‐binding cavity” (ABC) that intimately interacts with the substrate‐binding site (Figures 5, 6, and 7). The adenosyl group is held in the conserved and well‐structured ABC by nine conserved H‐bonds, two of which directly involve the protein backbone amide‐carbonyls of G68 and N123. Remarkably, the entirely structured (adenosine‐binding) cavity was also observed as a largely water‐filled space when vitamin B_12_ (CNCbl) or methylcobalamin (MeCbl) were bound in GM loaded with tartrate as pseudo‐substrate^[26]^ (see Supporting Information Figures S6 and S7). As shown here, the critical structural motif of adjacent adenosine‐ and substrate‐binding cavities of GM‐AdoCbl in the presence of the substrate glutamate or methylaspartate is precisely available also in the structures of GM reconstituted with the two “stretched” organometallic AdoCbl‐homologues AdoMeCbl and AdoEtCbl and in the presence of (*S*,*S*)‐tartrate as pseudo‐substrate. In GM‐AdoMeCbl, the cobalt‐bound AdoMe‐ligand fills the adenosine binding cavity with little strain, and its Co−C bond remains intact. The same is true for the AdoEt‐ligand in GM‐AdoEtCbl. Each Ado‐ligand (of AdoCbl, AdoMeCbl, and AdoEtCbl bound to GM) displaces a common set of four of the seven conserved water molecules found in the pre‐structured water‐filled ABC of GM‐CNCbl and GM‐MeCbl.^[26]^ In consequence, the Ado‐moieties of “activated” AdoCbl and of both the bound coenzyme B_12_ homologues AdoMeCbl and AdoEtCbl are positioned at the virtually same place and undergo the same H‐bonding interactions (Figure 6).

The three reconstituted (pseudo)‐substrate‐loaded GM structures harbour the Ado‐group above C5 (the northern *meso*‐position) of the corrin macrocycle and between its *a*‐ and *c*‐acetamide side chains, contrasting with the situation in crystalline AdoCbl^[24b]^ and AdoMeCbl,^[25a]^ where the respective adenosyl groups are located roughly above ring C in the southern hemisphere. As noted, the displaced adenosyl group of AdoCbl bound to substrate‐loaded GM (GM‐AdoCbl) displays two different ribose conformations, ^2^E (C2′‐endo) for the adenosyl group in the r_a_‐conformation and ^3^E (C3′‐endo) for its r_d_‐conformation. In crystals of AdoCbl^[24b]^ and AdoMeCbl,^[25a]^ the ribose unit also exists in ^3^E(C3′‐endo). However, in GM‐AdoMeCbl, the corresponding ribose unit of AdoMeCbl exhibits a ^2^T_3_ (2,3‐twist) conformation, similar to the particular ^2^E (C2′‐endo) r_a_‐conf in GM‐AdoCbl. In contrast, in GM‐AdoEtCbl, the organometallic AdoEt unit displays a ribose E_1_ (C1′‐exo) conformation. Hence, AdoMeCbl is bound to GM with not only an intact Co−C bond but also exhibits the typical hallmarks of a rather unstrained ribose moiety. In the case of AdoEtCbl, an intact Co−C bond is also observed, but the E_1_ (C1′‐exo) ribose conformation and a strained conformation of the linker between the adenine and the ethyl‐Cbl moieties are indicated. The experimental geometric parameters (see Supporting Information Table S2) reflect only a minor “pull” in the case of AdoMeCbl and an opposite small “squeeze” in AdoEtCbl. The structural restraints imposed by the protein‐part of GM on the bound Cbls (AdoCbl and its homologues) are largely relayed to the aliphatic “linker” connecting the corrin and the Ado‐moieties, which adapts to the small overall misfit in the two AdoCbl‐homologues. The organometallic linker takes up the strain that leads to Co−C bond rupture in the case of a bound functional B_12_‐cofactor AdoCbl, (see, e.g., Figure 5). The observation of intact Co−C bonds in the two “stretched” AdoCbl homologues bound to tartrate‐loaded GM confirms their designed basic capacity for structurally mimicking the key activated state(s) of GM‐bound “activated” AdoCbl,[^21^, ^25^] and is also consistent with biochemical experiments that showed no cofactor activity of AdoMeCbl in GM with either (*S*)‐glutamate or (2*S*,3*S*)‐3‐methylaspartate as substrates.^[27]^

In tartrate‐loaded GM‐AdoMeCbl and GM‐AdoEtCbl, the outer rim of ring B of the corrin macrocycle displays an exceptional eclipsed conformation. It is bent downwards, as also observed for substrate‐loaded GM‐bound AdoCbl (Supporting Information Figure S8). In each of the three structures, the corrin macrocycle is exceptionally flat with “corrin fold angles”^[28]^ of 2°, 4°, and 2°, respectively, which are remarkably smaller than in an imidazolyl‐cobamide model.^[29]^ This conformation allows for a critical intramolecular H‐bond between N3 of the adenine moiety of the organometallic β‐ligand and the c‐acetamide NH_2_ (Figure 4). This H‐bond and the concomitant flattening of the corrin ring are not seen in the GM structures reconstituted with vitamin B_12_ (GM‐CNCbl) or methylcobalamin (GM‐MeCbl),^[26]^ both lacking the adenosyl moiety (Supporting Information Figures S6 and S8). An intramolecular H‐bonding interaction in AdoCbl, but now of the adenosine ribose 3′‐OH with the a‐acetamide group, has likewise been deduced to be relevant for catalysis in diol dehydratase and ethanolamine ammonia‐lyase, two AdoCbl‐dependent eliminating isomerases.^[30]^

Our crystallographic studies of GM reveal critical structural details of the enzyme's active site, which houses adjacent and intimately cooperating adenosine‐ and substrate‐binding cavities. In the adenosine‐binding cavity (ABC), located roughly above the “northern” *meso*‐position of the corrin ring of AdoCbl, a precisely positioned Ado‐radical is generated with its H‐bonding adenine heterocycle clamped down tightly, allowing for only minor conformational mobility via pseudo‐rotation of its ribose unit.^[21]^ When the Ado‐radical reaches its previously discussed r_d_‐conformation, its C5′ is positioned in close proximity to the also tightly bound substrate molecules to achieve a precise H‐atom abstraction generating the firmly bound intermediate products 5′‐deoxyadenosine and substrate radical.^[21]^

Kinetic coupling between the homolysis of the Co−C bond and the generation of the 4‐glutamyl radical (or, alternatively, of a 3‐methyl‐3′‐aspartyl radical) has been deduced from studies of kinetic hydrogen‐isotope effects, compatible with reversible Co−C bond homolysis followed by a separate, rate‐determining H‐atom transfer.[^16a^, ^16b^, ^31^] The so‐generated 4‐glutamyl radical (or the alternative 3‐methyl‐3′‐aspartyl radical) is held tightly in the substrate‐binding cavity (SBC) by an “arginine claw” comprising the residues R66 and R149 and R100 (Figure 6 and Supporting Information Figure S9).^[21]^ The isomerisation step itself has been proposed to occur by a fragmentation/recombination mechanism involving a glycyl radical and acrylate as tightly bound intermediates, which is supported by experimental^[32]^ and computational^[33]^ evidence. The glycyl moiety is bound firmly by specific H‐bonding with the “upper part of the arginine claw” (R66, R149) and E171 (partial H‐bond to the glycyl amine). The hypothetical acrylate intermediate is deduced to be H‐bonded to R100 of the “lower part of the arginine claw”, as well as to Y177. It also makes several lipophilic contacts, one of these with H_3_C5′ of the inferred nearby bound 5′‐desoxyadenosine. The isomerisation occurs with retention of configuration at C2 of the glycyl moiety, and the comparison of the substrate and product structures implies a formal supra‐facial glycyl 1,2‐migration with respect to the acrylate moiety. Hence, a minor sliding of the acrylate relative to a firmly bound glycyl radical would fulfil the geometric requirements of the isomerisation. Indeed, a unique set of coherent and directed weakly bonding interactions in the substrate‐binding cavity provides a precisely structured tight radical reaction space for the interconverting substrate and product radicals and the hypothetical glycyl radical and acrylate fragments (Supporting Information Figure S9).

The collected crystallographic findings define a precisely structured active site in substrate‐loaded GM‐AdoCbl, which stabilises the bound highly activated forms of AdoCbl and binds enzyme substrates and products (and their hypothetical enzyme‐generated radicals) firmly. We posit the existence and key role of a functionalised caged radical reaction space (CRRS) in the active site of GM‐AdoCbl, a rigid protein module comprising the neighbouring ABC and SBC, assembled reversibly upon substrate binding and disassembled with product release (Figure 7).

The CRRS induces and harnesses the specific reversible radical chemistry of GM, guiding the enclosed radicals along a precisely confined trajectory without significant protein motion. It also ensures the efficient combination of the transient Ado‐radical with Cbl^II^ to complete the catalytic cycle and regenerate the cofactor AdoCbl, concurrent with product release. The Co^II^‐corrin Cbl^II^ is bound at the “lower” rim of the CRRS, exposing its reactive face inward for the rapid catch of the still elusive, confined Ado‐radical in a bonding process that requires little change of the cobalt‐corrin structure,^[15]^ thereby decisively reducing the lifetime of the highly reactive, spin‐coupled Ado‐radical.[^8^, ^34^] Otherwise, significant participation of Cbl^II^ is not indicated in the proper H‐atom transfer and radical isomerisation steps.

We conclude that in substrate‐loaded GM‐AdoCbl, the CRRS acts as a closed protein‐based two‐pronged vice (Figures 6 and 7, and Supporting Information Figure S9), supporting a firmly controlled, fast, and reversible reaction sequence involving highly reactive radicals, and employing AdoCbl as the reversibly functioning exquisite source of the Ado‐radical. The proper H‐atom transfer and isomerisation steps are amazing “least motion” processes of precisely and firmly positioned carbon‐centred radicals. The critical structural frame of a radical enzyme is described, which catalyses a carbon skeleton isomerisation with unique selectivity while inhibiting all competing side reactions, thus, perfectly achieving both “positive” and “negative” catalysis.[^3b^, ^3c^, ^35^] In consequence, besides the initial conformational “shuttling” of the Ado‐moiety of AdoCbl and the basically differing glutamate and 3‐methylaspartate skeletons, the crystallographic electron density does not help resolve the structures of the pairs glutamate vs. 4‐glutamyl radical or 3‐methylaspartate vs. 3‐methyl‐3′‐aspartyl radical. In fact, substrate or product molecules and their respective radical forms from H‐atom abstraction are pairwise alternating occupants of an indistinguishable part of the CRRS.

The crystal structures of tartrate‐loaded GM with AdoMeCbl or AdoEtCbl as pseudo‐cofactors and GM‐AdoCbl were assembled at room temperature but characterised crystallographically at cryo‐temperatures. The structure of substrate‐loaded GM‐AdoCbl^[21]^ is, thus, best seen as reflecting an immobilised “in‐action” form of a holo‐enzyme after turnover. A thorough EPR‐analysis of GM‐AdoCbl at 50 K revealed a very weakly spin‐coupled organic radical (characterised as the 4‐glutamyl radical) at a distance of about 6.6 Å from the Co^II^‐centre of Cbl^II^.^[22]^ Indeed, at steady state, GM features a relative spin concentration of up to 50 %, as observed by EPR.[^32b^, ^36^] Its UV/Vis‐spectra also show the complementary presence of a roughly comparable amount of Cbl^II^.[^10^, ^37^] The crystallographic and spectroscopic data are qualitatively consistent with a substrate‐ or product‐loaded GM‐AdoCbl, in which the “activated” AdoCbl exists in two roughly similarly populated states. One represents a crucially activated diamagnetic state of the B_12_‐cofactor in the enzyme, featuring a basically “stretched” Co−C bond and the Ado‐group in a r_a_‐conformation. The other one represents a “diradical state” with Cbl^II^ and either 5′‐deoxyadenosine largely as a direct neighbour of a 4‐glutamyl or 3‐methyl‐3′‐aspartyl radical, or else an Ado‐radical in the r_d_‐conformation in contact with glutamate or 3‐methylaspartate. The hardly distinguished electron density between glutamate and 4‐glutamyl radical or between 3‐methylaspartate and 3‐methyl‐3′‐aspartyl radical is consistent with the absence of significant pairwise differences in their inherent structures and their intermolecular polar interactions (see Supporting Information Figure S10). Hence, we propose the crystallographic r_d_‐conformation of the Ado group to predominantly represent 5′‐deoxyadenosine (rather than the elusive free Ado‐radical) in contact with a 4‐glutamyl radical or with a 3‐methyl‐3′‐aspartyl radical. In this scenario, C4 of the bound 4‐glutamyl radical would contact C5′ of deoxyadenosine (at a distance of 3.3 Å) and would be positioned at approximately 7 Å from the cobalt centre of Cbl^II^. Likewise, C3′ of the alternatively bound 3‐methyl‐3′‐aspartyl radical, which displays a short and presumably weakly stabilising contact of 3.0 Å to the phenolic oxygen of Y181, is at a distance of 3.0 Å from C5′ of deoxyadenosine and approximately 6.6 Å from the cobalt centre of Cbl^II^. The crystallographically deduced distances and the complementary observation of spin‐spin interactions between a 4‐glutamyl radical and Cbl^II^ by EPR are compatible with far‐reaching Co^II^‐based weak interactions with radical intermediates,^[22]^ which may join the array of conserved H‐bonds (see Figure 6) to help “conduct” the trajectory of the Ado‐radical during the GM‐reaction.[^13c^, ^34^, ^38^]

**Figure 8:**
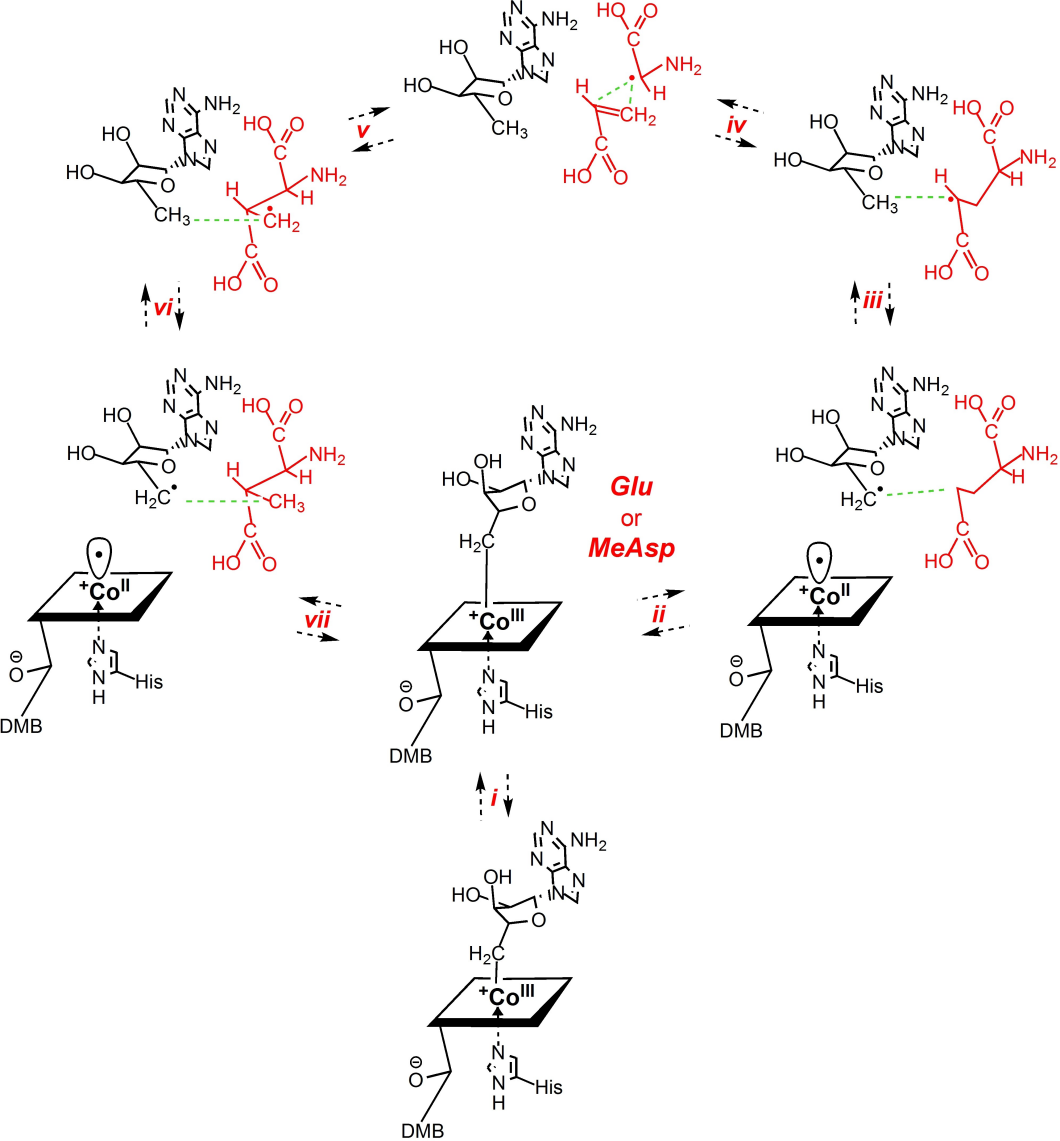
Detailed structure‐based mechanism of GM induced by binding of the enzyme substrates (*S*)‐glutamate (Glu) or (2*S*,3*S*)‐3‐methylaspartate (MeAsp). In the substrate‐free “resting state” of GM‐AdoCbl, AdoCbl bound between the protein ϵ‐ and σ‐subunits is proposed to have its Ado‐moiety moved into a large protein pocket above the northern *meso*‐position of the corrin ligand. Substrate binding (*i*) restructures the protein, generates a tight adenosine‐binding pocket as part of a caged radical reaction space, and induces the stretch of the Co−C5′ bond to 3.2 Å, the r_a_‐conformation. In the “forward” direction shown counterclockwise in this figure, ribose‐pseudo‐rotation and radical shuttling to the r_d_‐conformation (*ii*) put C5′ close to the substrate and at 4.2–4.5 Å from cobalt, breaking the Co−C5′ bond. The Ado‐radical abstracts an H‐atom from the 4‐position of Glu (*iii*) quickly in a thermodynamically favourable step. Radical C−C cleavage (*iv*) generates an acrylate fragment and the bound glycyl‐radical. Subsequent radical C−C recombination (*v*) enables the overall migration of the acrylate fragment relative to the bound glycyl‐radical, producing the primary 3‐methyl‐3′‐aspartyl radical. The bound primary 3‐methyl‐3′‐aspartyl radical re‐abstracts an H‐atom from the closely positioned C5′ of deoxyadenosine (*vi*). The so‐formed Ado‐radical, generated in the r_d_‐conformation, shuttles back to the r_a_‐conformation, furnishing a weak Co−C bond from attraction by Cbl^II^ (*vii*). Reconstitution of the intact AdoCbl drives the disassembly of the Ado‐binding pocket and product release, regenerating GM‐AdoCbl in its “resting state”.

For the catalysis by GM, we can now assign a firm structural frame to the complete sequence of fully reversible reaction steps, beginning with the substrate‐induced Co−C bond homolysis of the bound AdoCbl and involving subsequently generated radicals confined and controlled along their reaction trajectory by a rigid protein cage (see Figure 8).

Substrate loading of GM structures a “Procrustean” bed for the bound AdoCbl, imposing a decisive misfit on it and causing elongation and homolysis of its Co−C bond. This model assigns the structural basis of the roughly 10^12^‐times acceleration of the homolysis of the Co−C bond in GM‐AdoCbl (overall rates of the isomerisation by GM: 16–20 s^−1^)^[10]^ to the misfit of AdoCbl to the substrate‐loaded protein and a stabilising binding complement for the separated AdoCbl‐homolysis fragments. Hence, the required thermodynamic driving force for the relative stabilisation of the bound “activated” AdoCbl is provided by binding its detached Ado‐moiety in the rigid ABC, which is structured precisely upon substrate incorporation into the adjacent substrate‐binding half‐space of the CRRS. In contrast, when AdoCbl is bound to GM without substrate, it is not activated to a significant extent, its UV/Vis‐spectrum is not altered significantly, and radicals are not generated.[^37^, ^39^] When, in the substrate‐free GM‐AdoCbl, the Ado‐radical and Cbl^II^ are produced artificially by photolytic homolysis of the Co−C bond of AdoCbl, the homolysis fragments recombine exceedingly fast and nearly quantitatively,^[16c]^ consistent with an Ado‐radical confined in close proximity to the Cbl^II^‐fragment of AdoCbl. Hence, an essentially un‐activated B_12_‐cofactor exists in substrate‐free GM, for which a crystal structure is, unfortunately, not available. However, its Ado‐ligand is proposed to be reoriented into a weakly interacting adenosine‐binding region at the subunit interface, placing the Ado‐ligand roughly above the “northern” *meso*‐position of the corrin ring of AdoCbl (see Supporting Information Figure S11). This provisional conclusion for substrate‐free GM‐AdoCbl is deduced from analogy with the arrangement of the Ado‐moiety in the related case of substrate‐free MCM characterised crystallographically (see below).[^6d^, ^23^]

Indeed, roughly similar architectural topologies may exist for the radical reaction space in the two AdoCbl‐dependent carbon skeleton mutases GM and MCM.[^6b^, ^11^] In MCM without substrate, a large cavity is observed above the “northern” *meso*‐position of the bound B_12_ (see Supporting Information Figure S12),[^6d^, ^19^, ^23^] corresponding to the position of the adenosine‐binding site seen in substrate‐loaded MCM^[19]^ and GM.^[21]^ However, specific binding interactions between the adenosine moiety and the protein cannot be recognised, aside from one weak H‐bond with the ribose‐2′‐OH.^[6d]^ Thus, the bound AdoCbl is first prepared for its eventual job in the ABC by reorienting the Ado‐ligand towards the north, but without its binding in an activated state. In contrast, when the substrate methylmalonyl‐CoA is bound, a well‐structured adenosine‐binding cavity is available in the TIM‐barrel domain of MCM, located directly above the “northern” *meso*‐position of the corrin ring of the bound AdoCbl (see Supporting Information Figure S13). Binding of the substrate methylmalonyl‐CoA clamps down the Ado‐ligand of AdoCbl and activates it for Co−C bond homolysis.[^6d^, ^19^, ^23^] For MCM, a crucial conformational repositioning of the Ado‐moiety has been deduced,[^6d^, ^23^] comparable to the “radical shuttling” observed in GM,^[21]^ and the addition of the intermediary Ado‐radical to the inhibitory metabolite itaconate has allowed trapping of the de‐routed enzyme in a stable diradical state.^[6d]^ The available insights into the mode of action of MCM[^6b^, ^6d^] indicate a strongly related strategy displayed by the AdoCbl‐dependent carbon skeleton mutases GM and MCM, as is the case for other acyl‐CoA mutases meanwhile characterised by crystallography (see Supporting Information Figure S13).^[20]^

As reported, the critical structural features of GM and its caged radical reaction space may, hence, serve as models for the group of mechanistically related carbon skeleton mutases. The likewise important AdoCbl‐dependent eliminating isomerases, such as diol dehydratase (DD) and ethanolamine ammonia‐lyase (EAL),[^6c^, ^30^, ^40^] use a remarkably wider space for their AdoCbl‐initiated radical processes. When the “stretched” B_12_‐homologues AdoMeCbl and AdoEtCbl were tested with DD and EAL, AdoEtCbl showed no activity with either enzyme, as expected, whereas, surprisingly, AdoMeCbl acted as a very inefficient and rapidly enzyme‐deactivating B_12_‐pseudocofactor in both enzyme assays.^[41]^ Ongoing crystallographic studies by N. Shibata, T. Toraya et al.^[42]^ of the two eliminating isomerases with bound AdoMeCbl are expected to provide insights into the dynamics of the radical reaction of this class of enzymes and help clarify their striking residual activity with the “stretched” AdoCbl‐homologue AdoMeCbl.

## Conclusion

A comprehensive structure‐ and mechanism‐based frame for the GM reaction is presented. Our data with GM define a rigidly structured, caged radical reaction space (CRRS). It is the combination of interacting adenosine‐ and substrate‐binding protein cavities confining and controlling this enzyme's entire cascade of radical intermediates. In this environment, the innate chemistry of the organometallic AdoCbl ensures the thermodynamic and kinetic feasibility of the radical generation and a high degree of its reversibility. Indeed, AdoCbl is a uniquely suitable organometallic pre‐catalyst, featuring the characteristics of a reversible source of the reactive Ado‐radical.[^7^, ^13c^, ^43^] The critical driving force for the substrate‐induced activation of the Co−C bond homolysis of the base‐off/His‐on bound AdoCbl is provided by an excellent binding complement for the largely separated homolysis fragments, Ado‐radical and Cbl^II^, and the corresponding structural misfit of the protein for the intact B_12_‐cofactor, consistent with our earlier postulate.^[15]^ As precisely modelled by the homologues of AdoCbl, the substrate‐loaded protein activates the bound AdoCbl specifically towards the elongation and cleavage of its Co−C bond, liberating the primary Ado‐radical. This radical initiates the key reversible H‐atom abstraction and substrate radical isomerisation steps and returns with high fidelity (like a molecular boomerang) to the persistent radical trap Cbl^II^ for regeneration of AdoCbl. The Ado‐radical carries the functional handles for its tight control by protein‐binding and an intricate reactivity as a primary radical for quick H‐atom abstraction,[^8^, ^9^] as required for the carbon skeleton mutases.[^6a^, ^13c^] Furthermore, the rigid CRRS is assembled upon firm substrate binding as the crucial internal protein module that provides a tight grip for the reacting radicals and limits their reaction space to the trajectory required by the highly selective process catalysed by GM. Combining the unique inherent reactivity of Nature's most intriguing cofactor^[44]^ and the principles of “negative catalysis”,^[3b]^ AdoCbl‐dependent carbon skeleton mutases excel by their capacity for efficiently catalysing many turnovers of most “difficult chemistry”, using firmly bound radicals confined to a rigidly structured caged reaction space.

## Experimental Section

The structures of GM reconstituted with AdoMeCbl and AdoEtCbl were determined by x‐ray crystallography using synchrotron radiation to resolutions of 1.8 and 2.1 Å, respectively. For details of the data collection and structure refinement, see the Materials and Methods section in the Supporting Information. Coordinates and structure factors were deposited in the Protein Data Bank (PDB) under accession numbers 6H9E (GM‐AdoMeCbl) and 6H9F (GM‐AdoEtCbl).

## Conflict of interest

The authors declare no conflict of interest.

1

## Supporting information

As a service to our authors and readers, this journal provides supporting information supplied by the authors. Such materials are peer reviewed and may be re‐organized for online delivery, but are not copy‐edited or typeset. Technical support issues arising from supporting information (other than missing files) should be addressed to the authors.

Supporting Information

## Data Availability

Coordinates and structure factors have been deposited in the Protein Data Bank (PDB) under accession numbers 6H9E and 6H9F.
